# Co-Deletion of Chromosome 1p/19q and IDH1/2 Mutation in Glioma Subsets of Brain Tumors in Chinese Patients

**DOI:** 10.1371/journal.pone.0032764

**Published:** 2012-03-12

**Authors:** Xiaohui Ren, Xiangli Cui, Song Lin, Junmei Wang, Zhongli Jiang, Dali Sui, Jing Li, Zhongcheng Wang

**Affiliations:** 1 Neurosurgery, Capital Medical University, Beijing Tiantan Hospital, Beijing, China; 2 Beijing Neurosurgical Institute, Beijing, China; University of Chicago, United States of America

## Abstract

**Objective:**

To characterize co-deletion of chromosome 1p/19q and IDH1/2 mutation in Chinese brain tumor patients and to assess their associations with clinical features.

**Methods:**

In a series of 528 patients with gliomas, pathological and radiological materials were reviewed. Pathological constituents of tumor subsets, incidences of 1p/19q co-deletion and IDH1/2 mutation in gliomas by regions and sides in the brain were analyzed.

**Results:**

Overall, 1p and 19q was detected in 339 patients by FISH method while the sequence of IDH1/2 was determined in 280 patients. Gliomas of frontal, temporal and insular origin had significantly different pathological constituents of tumor subsets (*P*<0.001). Gliomas of frontal origin had significantly higher incidence of 1p/19q co-deletion (50.4%) and IDH1/2 mutation (73.5%) than those of non-frontal origin (27.0% and 48.5%, respectively) (*P*<0.001), while gliomas of temporal origin had significantly lower incidence of 1p/19q co-deletion (23.9%) and IDH1/2 mutation (41.7%) than those of non-temporal origin (39.9% and 63.2%, respectively) (*P* = 0.013 and *P* = 0.003, respectively). Subgroup analysis confirmed these findings in oligoastrocytic and oligodendroglial tumors, respectively. Although the difference of 1p/19q co-deletion was not statistically significant in temporal oligodendroglial tumors, the trend was marginally significant (*P* = 0.082). However, gliomas from different sides of the brain did not show significant different pathological constituents, incidences of 1p/19q co-deletion or IDH1/2 mutation.

**Conclusion:**

Preferential distribution of pathological subsets, 1p/19q co-deletion and IDH1/2 mutation were confirmed in some brain regions in Chinese glioma patients, implying their distinctive tumor genesis and predictive value for prognosis.

## Introduction

Glioma is the most common primary intracranial tumor [1]. According to 2007 WHO Classification of Tumors of the Central Nervous System [2], gliomas consist of all brain tumors that are of glial cell origin, accounting for almost 80% of primary malignant brain tumors [3]. They are graded according to biological behavior of the tumors. With the exception of pilocytic astrocytomas, the prognosis of glioma patients is still poor, though combined modality therapy and individualized treatment have been widely used [1].

Co-deletion of chromosome 1p/19q, as a recognized prognostic predictor, is associated with a good prognosis and increased responsiveness to chemotherapy. High incidence of 1p and 19q deletion is observed in oligodendroglioma and oligoastrocytoma [4]. Co-deletion of 1p and 19q is associated with longer progression-free time and longer median survival time, thus representing an independent prognostic factor in anaplastic oligodendroglial tumors (WHO grade III) [5], [6], [7], [8]. Similarly, 1p/19q co-deletion also predicts a longer radiographic response to temozolomide and is associated with both superior overall survival and progression-free survival in low-grade oligodendroglial tumors [9], [10], [11], [12].

The discovery of somatic mutations in the isocitrate dehydrogenase (IDH) enzymes through a genome-wide mutational analysis in glioblastoma represents a milestone event in cancer biology [13]. Recent reports showed IDH1 mutations in astrocytic and oligodendroglial tumors (WHO grade II and III) and in secondary glioblastomas with a frequency of up to 90%, whereas IDH1 mutations occurred in only 5% of primary glioblastomas [14]. Patients with diffusely infiltrative gliomas carrying an IDH1 mutation have a significantly longer overall survival compared to patients with IDH1 wild type [15], [16]. IDH1 codon 132 mutations were found to be associated with reduced NADP-dependent IDH activity in glioblastoma [16]. The low NADPH levels may sensitize glioblastoma to irradiation and chemotherapy, thus explaining the prolonged survival of patients with mutated glioblastoma [17].

Recently, several studies implied that gliomas located at different part of the brain might harbor individualized pathological constituents. Larjavaara et al. demonstrated considerable heterogeneity in the anatomic distribution of gliomas within the brain [18]. Duffau et al. reported that low-grade gliomas were located preferentially in the “secondary” functional areas, especially within the region of the supplementary motor area and the insular lobe [19]. Besides, some studies identified an association between the incidence of 1p/19q co-deletion, IDH1/2 mutation and tumor locations. Zlatescu et al. suggested that tumor genotypes were closely associated with tumor locations [20], because they found that anaplastic oligodendrogliomas located in the frontal, parietal, and occipital lobes were significantly more likely to harbor allelic loss of chromosome 1p and 19q than histologically indistinguishable tumors arising in the temporal lobe, insula, and diencephalon; in addition, loss of heterozygosity (LOH) for 1p and 19q was significantly associated with a bilateral pattern of growth. Mueller et al. reported that allelic loss of 1p and 19q was significantly less frequent in temporal oligodendrogliomas and oligoastrocytomas [21]. Huang et al. reported that oligodendroglial tumors having LOH of 1p and 19q occurred most frequently in the non-temporal lobes [22]. Goze et al. reported a relatively lower incidence of allelic loss of chromosome 1p and 19q in a consecutive series of 12 Grade II gliomas involving the insula [23]. However, Wu et al. reported a high rate of 1p/19q co-deletion in insular oligodendroglial tumors [24]. It was also found that IDH1 mutant glioblastomas predominantly involved the frontal lobe [25].

To investigate whether subsets, 1p/19q co-deletion and IDH1/2 mutation of the tumors are correlated with tumor locations in Chinese glioma patients, we analyzed a series of 528 gliomas for the correlation of their pathological constituents of subsets, the incidence of 1p/19q co-deletion and IDH1/2 mutation with tumor locations.

## Materials and Methods

### Ethics statement

All patients provided written informed consent for the current study and the clinical study was approved by the Medical Ethics Committee of Capital Medical University.

### Patients and tissue samples

A series of 528 primary glioma patients, including 308 male and 220 female, were surgically treated in the Department of Supratentorial Neoplasms of Beijing Tiantan Hospital between January 2008 and September 2010. The patients' ages ranged from 14 to 68 years old with a mean of 41.5±11.6 years. This series of 528 gliomas consisted of 108 astrocytoma WHO grade II (A), 45 anaplastic astrocytoma WHO grade III (AA), 88 primary glioblastoma WHO grade IV (G), 80 oligodendroglioma WHO grade II (O), 37 anaplastic oligodendroglioma WHO grade III (AO), 99 oligoastrocytoma WHO grade II (OA) and 71 anaplastic oligoastrocytoma WHO grade III (AOA). All glioma cases enrolled in the Department of Neuropathology, Beijing Neurosurgical Institute, were examined and graded independently by two neuropathologists who were blind to tumor genotypes, according to the 2007 World Health Organization (WHO) Classification of Tumors of the Central Nervous System [2]. Histological diagnoses of tumor specimens were reviewed and confirmed by a third neuropathologist. If the first two pathologists did not agree on the diagnosis, a third senior neuropathologist would make the judge. If the three neuropathologists could not make agreement, this case would be submitted to the pathological committee of Beijing Neurosurgical Institute and Beijing Tiantan Hospital for final diagnosis. The committee was composed of 10 senior neuropathologists and final diagnosis would be made only when more than 6 of them agreed on it.

### Detection of deletion of 1p/19q by the fluorescence in situ hybridization (FISH) method

FISH was used for the detection of 1p/19q deletion in a series of 339 gliomas as a routine test from Jan 2009, including 150 astrocytic, 111 oligoastrocytic, and 78 oligodendroglial tumors.

The 1p/19q fluorescent probe kit (Vysis, USA) was used for the FISH test. Breifly, 4 µm thick paraffin slides were deparaffinized, dehydrated, and incubated in 1 mol/L NaSCN for 35 min at 80°C. Slides were then immersed in pepsin solution (0.65% in protease buffer with 0.01 mol/L HCl) for 10 min at 37°C, and tissues were fixed by 10% neutral buffered formalin. Then the specimens were dehydrated in ethanol (70%, 85%, and 100%, 2 min in each bath) and air-dried, 20 ul of each probe was then added separately, and slides were sealed with rubber cement. After co-denaturation for 10 min at 75°C, the slides were then put in a humidified atmosphere with Hybrite (ThermoBrite™ vysis) 16 h at 37°C. Slides were immersed first in 2× SSC/0.3% NP-40 for 2 min at RT and then in 2×SSC/0.3% NP-40 for 2 min at 73°C. After drying, nuclei were counterstained with 4, 6-diamidino- 2-phenylindole (DAPI) and antifade compound (Pphenylenediamine). FISH signals for each locus-specific FISH probe were assessed under an Olympus BX51TRF microscope (Olympus, Ina-shi, Nagano, Japan) equipped with a triple-pass filter (DAPI/Green/Orange; Vysis). The assessment and interpretation of FISH results were made according to guidelines defined by the SIOP Europe Neuroblastoma Pathology and Biology and Bone Marrow Group [26]. For each probe, more than 100 non overlapping nuclei were enumerated per hybridization. Tumors with more than 30% of nuclei showing DNA loss were defined as tumor with chromosomal loss.

### IDH1/2 sequence analysis

IDH1 and IDH2 genes were sequenced in 280 gliomas, including 105 astrocytic, 104 oligoastrocytic, and 71 oligodendroglial tumors.

Genomic DNA was isolated from snap-frozen tissue using the QIAmp DNA mini-kit, as described by the manufacturer (Qiagen). A fragment of 254 bp length spanning the catalytic domain of IDH1 including codon 132 was amplified using the sense primer IDH1 F: 5′-ACCAAATGGCACCATACG-3′ and the antisense primer IDH1 R: 5′-TTCATACCTTGCTTAATGGGG-3′. A fragment of 293 bp length spanning the catalytic domain of IDH2 including codon 172 was amplified using the sense primer IDH2 F: 5′-GCTGCAGTGGGACCACTATT-3′ and the antisense primer IDH2 R: 5′-TGTGGCCTTGTACTGCAGAG ′. PCR using standard buffer conditions, 30 ng of DNA and GoTaq DNA Polymerase (TaKaRa, Japan) employed 35 cycles with denaturing at 95°C for 30 s, annealing at 54°C for 45 s and extension at 72°C for 50 s in a total volume of 25 µL. The PCR amplification product was sent to Beijing Tianyi Huiyuan Bioscience and Technology Incorporation for sequencing.

### Tumor location

Tumor location was considered to be the lobe or region of the brain, in which the bulk of the tumor resided. They were specified according to the International Classification of Diseases, version 10 (ICD-10) as previously described by Larjavaara [18]. Besides, we added additional two locations: gliomas in the insular lobe were assigned according to the classification of tumors of limbic and pralimbic systems by Yasargil [27] and gliomas in thalamus, basal ganglia or ventricle were defined as deeply located tumors. These assignments were based on the evaluation of the MR images, including T1-weighted axial, coronary, and sagital images before and after Gadolinium enhancement and T2-weighted axial images.

### Statistical analysis

Chi-square (χ^2^) test was used for the comparison of differences among regional constituents of pathological subsets, regional percentages of tumor subsets and regional incidences of 1p/19q co-deletion and IDH1/2 mutation. SPSS 13.0 (SPSS for Windows, version 13.0 [SPSS Inc., Chicago, Illinois, USA]) was used for statistical analysis. Probability value was obtained from 2-sided tests, with a statistical significance of *P*<0.05.

## Results

### Regional constituents of tumor pathologies (Figure 1)

**Figure 1:**
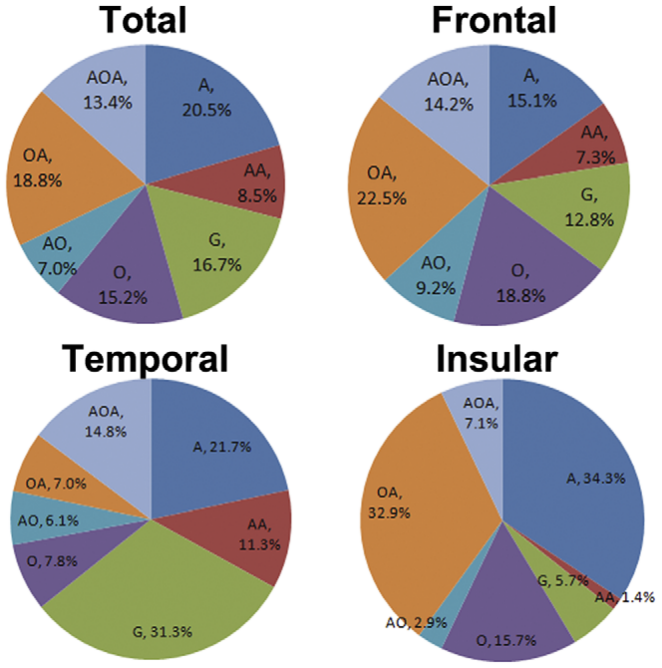
Gliomas of frontal, temporal and insular origin had significantly different pathological constituents of tumor subsets. The percentage of astrocytoma in gliomas of the frontal origin (15.1%) was significantly lower than that of non-frontal origin (24.2%) (*P* = 0.011). The percentage of glioblastoma in gliomas of the frontal origin (12.8%) was significantly lower than that of non-frontal origin (19.4%) (*P* = 0.048). The percentage of oligodendroglioma in gliomas of the frontal origin (18.8%) was significantly higher than that of non-frontal origin (12.6%) (*P* = 0.049). The percentage of glioblastoma in gliomas of the temporal origin (31.3%) was significantly higher than that of non-temporal origin (12.6%) (*P*<0.001). The percentage of oligodendroglioma in gliomas of the temporal origin (7.8%) was significantly lower than that of non-temporal origin (17.2%) (*P* = 0.013). The percentage of oligoastrocytoma in gliomas of the temporal origin (7.0%) was significantly lower than that of non-temporal origin (22.0%) (*P*<0.001). The percentage of astrocytoma in gliomas of the insular origin (34.3%) was significantly higher than that of non-insular origin (18.3%) (*P* = 0.002). The percentage of oligoastrocytoma in gliomas of insular origin (32.9%) was significantly higher than that of non-insular origin (16.6%) (*P* = 0.001). The percentage of anaplastic astrocytoma in gliomas of the insular origin (1.4%) was significantly lower than that of non-insular origin (9.6%) (*P* = 0.022). The percentage of glioblastoma (5.7%) was significantly lower than that of non-insular origin (18.3%) (*P* = 0.008). A = astrocytoma; AA = anaplastic astrocytoma; G = glioblastoma; O = oligodendroglioma; AO = anaplastic oligodendroglioma; OA = oligoastrocytoma; AOA = anaplastic oligoastrocytoma.

Pathological constituents of gliomas of the frontal origin (A 15.1%, AA 7.3%, G 12.8%, O 18.8%, AO 9.2%, OA 22.5%, and AOA 14.2%) was significantly different from that of non-frontal origin (A 24.2%, AA 9.4%, G 19.4%, O 12.6%, AO 5.5%, OA 16.1% and AOA 12.9%) (*P* = 0.007). The percentage of astrocytoma in gliomas of the frontal origin (15.1%) was significantly lower than that of non-frontal origin (24.2%) (*P* = 0.011). The percentage of glioblastoma in gliomas of the frontal origin (12.8%) was significantly lower than that of non-frontal origin (19.4%) (*P* = 0.048). The percentage of oligodendroglioma in gliomas of the frontal origin (18.8%) was significantly higher than that of non-frontal origin (12.6%) (*P* = 0.049). The percentage of oligoastrocytoma in gliomas of the frontal origin (22.5%) showed a trend to be higher than that of non-frontal origin (16.1%), but the difference did not reach statistical significance (*P* = 0.066).

Pathological constituents of gliomas of the temporal origin (A 21.7%, AA 11.3%, G 31.3%, O 7.8%, AO 6.1%, OA 7.0%, and AOA 14.8%) was significantly different from that of non-temporal origin (A 20.1%, AA 7.7%, G 12.6%, O 17.2%, AO 7.3%, OA 22.0% and AOA 13.1%) (*P*<0.001). The percentage of glioblastoma in gliomas of temporal origin (31.3%) was significantly higher than that of non-temporal origin (12.6%) (*P*<0.001). The percentage of oligodendroglioma in gliomas of the temporal origin (7.8%) was significantly lower than that of non-temporal origin (17.2%) (*P* = 0.013). The percentage of oligoastrocytoma in gliomas of the temporal origin (7.0%) was significantly lower than that of non-temporal origin (22.0%) (*P*<0.001).

Pathological constituents of gliomas of the insular origin (A 34.3%, AA 1.4%, G 5.7%, O 15.7%, AO 2.9%, OA 32.9% and AOA 7.1%) was significantly different from that of non-insular origin (A 18.3%, AA 9.6%, G 18.3%, O 15.1%, AO 7.6%, OA 16.6% and AOA 14.4%) (*P*<0.001). The percentage of astrocytoma in gliomas of the insular origin (34.3%) was significantly higher than that of non-insular origin (18.3%) (*P* = 0.002). The percentage of oligoastrocytoma in gliomas of the insular origin (32.9%) was significantly higher than that of non-insular origin (16.6%) (*P* = 0.001). The percentage of anaplastic astrocytoma in gliomas of the insular origin (1.4%) was significantly lower than that of non-insular origin (9.6%) (*P* = 0.022). The percentage of glioblastoma (5.7%) was significantly lower than that of non-insular origin (18.3%) (*P* = 0.008). The percentage of anaplastic oligoastrocytoma (7.1%) showed a trend to be lower than that of non-insular origin (14.4%), but the difference did not reach statistical significance (*P* = 0.097).

The percentage of astrocytoma in gliomas of the occipital origin (41.2%) showed a trend to be higher than that of non-occipital origin (19.8%), but the difference did not reach significance (*P* = 0.065). The percentage of astrocytoma in deeply located gliomas (33.3%) showed a trend to be higher than that of other origin (19.8%), but the difference did not reach significance either (*P* = 0.089).

Gliomas of frontal, temporal and insular origins harbored significantly different pathological constituents (*P*<0.001), whereas the pathological constituents of gliomas in the left, right and bilateral sides were not significantly different (*P* = 0.589).

### Incidences of 1p/19q co-deletion and IDH1/2 mutation in different regions and sides of the brain, irrespective of pathology (Table 1 and Figure 2 and 3)

**Figure 2:**
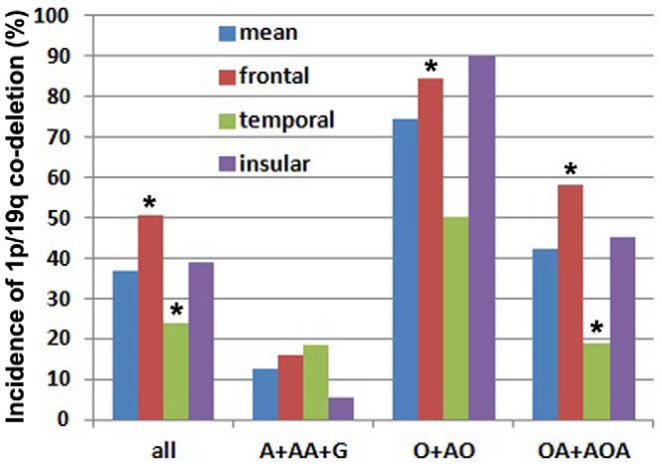
Gliomas of frontal and temporal origin had significantly different incidences of 1p/19q co-deletion irrespective and in respective of tumor pathology. The regional incidences of 1p/19q co-deletion in all gliomas of the region irrespective of pathology were labeled with “All”. The regional incidences of 1p/19q co-deletion in respective of pathology were labeled with “A+AA+G, O+AO and OA+AOA” respectively. *The incidence of 1p/19q co-deletion in this region is significantly higher or lower than that in other regions (*p*<0.05, chi-square test).

**Figure 3:**
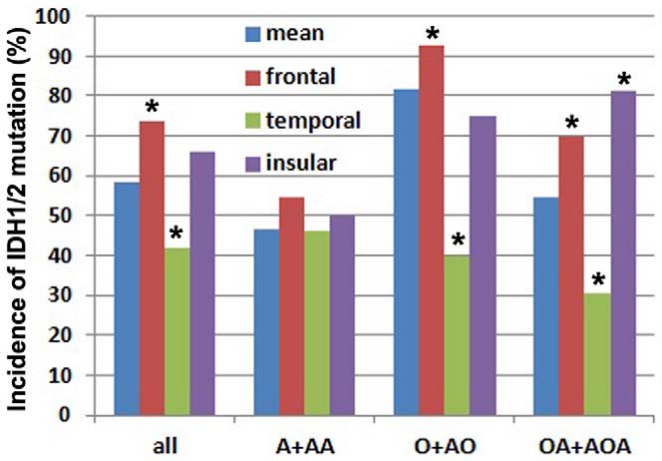
Gliomas of frontal and temporal origin had significantly different incidences of IDH1/2 mutation irrespective and in respective of tumor pathology. The regional incidences of IDH1/2 mutation in all gliomas of the region irrespective of pathology were labeled with “All”. The regional incidences of IDH1/2 mutation in respective of pathology were labeled with “A+AA, O+AO and OA+AOA” respectively. *The incidence of IDH1/2 mutation in this region is significantly higher or lower than that in other regions (p<0.05, chi-square test).

**Table 1:** Regional frequencies of 1p/19q co-deletion and IDH1/2 mutation in glioma subsets.

			Frequency in different lobes							Frequency in different sides		
Pathology	Molecular alteration	Frequency	Frontal	Temporal	Insular	Parietal	Occipital	Fronto-parietal	Deeply located	Left	Right	Bilateral
O+AO	1p/19q co-deletion	58/78	33/39	6/12	9/10	6/10	-	2/4	2/3	30/39	24/35	4/4
O+AO	IDH1/2 mutation	58/71	38/41	4/10	6/8	7/8	-	3/3	0/1	28/33	25/33	5/5
OA+AOA	1p/19q co-deletion	47/111	29/50	3/16	9/20	3/13	1/1	2/8	0/3	24/51	19/52	4/8
OA+AOA	IDH1/2 mutation	57/104	28/40	7/23	13/16	6/13	0/2	2/7	1/3	27/47	27/54	3/3
A+AA+G	1p/19q co-deletion	19/150	8/50	8/43	1/19	1/10	0/8	1/11	0/9	10/65	8/74	1/11
A+AA	IDH1/2 mutation	49/105	17/32	14/27	8/17	0/9	5/6	1/4	4/10	20/47	26/48	3/10
Total	1p/19q co-deletion	124/339	70/139	17/71	19/49	10/33	1/9	5/23	2/15	64/155	51/161	9/23
	IDH1/2 mutation	164/280	83/113	25/60	27/41	13/30	5/8	6/14	5/14	75/127	78/135	11/18

A: astrocytomas; AA: anaplastic astrocytoma; G: glioblastoma; O: oligodendrogliomas; AO: anaplastic oligodedroglioma; OA: oligoastrocytomas; AOA: anaplastic oligoastrocytoma; IDH: isocitrate dehydrogenase gene; (-): no data available. Numbers of cases with alterations are given in respect to cases examined.

In 339 gliomas, the mean global incidence of 1p/19q co-deletion was 36.6%. Irrespective of pathology, the incidence of 1p/19q co-deletion in gliomas of the frontal origin (50.4%) was significantly higher than that of non-frontal origin (27.0%) (*P*<0.001). The incidence of 1p/19q co-deletion in gliomas of the temporal origin (23.9%) was significantly lower than that of non-temporal origin (39.9%) (*P* = 0.013). The incidence of 1p/19q co-deletion in deeply located gliomas (13.3%) showed a trend to be lower than that of non-deeply located gliomas (37.7%), but the difference did not reach statistical significance (*P* = 0.056). Furthermore, the incidences of 1p/19q co-deletion in the left, right and bilateral sides of the brain (41.3%, 31.7% and 39.1%, respectively) did not show a significant difference (*P* = 0.200).

In 280 gliomas, the mean global incidence of IDH1/2 mutation was 58.6%. Irrespective of pathology, the incidence of IDH1/2 mutation in gliomas of the frontal origin (73.5%) was significantly higher than that of non-frontal origin (48.5%) (*P*<0.001). The incidence of IDH1/2 mutation in gliomas of the temporal origin (41.7%) was significantly lower than that of non-temporal origin (63.2%) (*P* = 0.003). The incidence of IDH1/2 mutation in gliomas of parietal origin (43.3%) showed a trend to be lower than that of non-parietal origin (60.4%) (*P* = 0.073). Furthermore, the incidence of IDH1/2 mutation in deeply located gliomas (35.7%) showed a trend to be lower than that of non-deeply located gliomas (59.8%) (*P* = 0.075). The incidences of IDH1/2 mutation in the left, right and bilateral sides of the brain (59.1%, 57.8% and 61.1%, respectively) did not show a significant difference (*P* = 0.954).

### Regional incidences of co-deletion of chromosome 1p/19q and IDH1/2 mutation in subgroups (Table 1 and Figure 2 and 3)

In 111 oligoastrocytic tumors (Grade II and III), the mean global incidence of 1p/19q co-deletion was 42.3%. The incidences in Grade II and Grade III (43.1% and 41.3%, respectively) were not significantly different (*P* = 0.852). The incidence of 1p/19q co-deletion in oligoastrocytic tumors of the frontal origin (58.0%) was significantly higher than that of non-frontal origin (29.5%) (*P* = 0.003). The incidence of 1p/19q co-deletion in oligoastrocytic tumors of the temporal origin (18.8%) was significantly lower than that of non-temporal origin (46.3%) (*P* = 0.039). For oligoastrocytic tumors, however, the incidences of 1p/19q co-deletion between different sides of the brain (47.1%, 36.5% and 50.0%, respectively) were not significantly different.

In 104 oligoastrocytic tumors (Grade II and III), the mean global incidence of IDH1/2 mutation was 54.8%. The incidences in Grade II and Grade III were not significantly different (*P* = 0.340). The incidence of IDH1/2 mutation in oligoastrocytic tumors of the frontal origin (70.0%) was significantly higher than that of non-frontal origin (45.3%) (*P* = 0.014). The incidence of IDH1/2 mutation in oligoastrocytic tumors of the temporal origin (30.4%) was significantly lower than that of non-temporal origin (61.7%) (*P* = 0.008). The incidence of IDH1/2 mutation in oligoastrocytic tumors of the insular origin (81.3%) was significantly higher than that of non-insular origin (50.0%) (*P* = 0.021). For oligoastrocytic tumors, however, the incidences of IDH1/2 mutation between different sides of the brain (57.4%, 50.0% and 100.0%, respectively) were not significantly different.

In 78 oligodendroglial tumors (Grade II and III), the mean global incidence of 1p/19q co-deletion was 74.4%. The incidences in Grade II and Grade III (78.6% and 63.6%, respectively) were not significantly different (*P* = 0.174). The incidence of 1p/19q co-deletion in oligodendroglial tumors of the frontal origin (84.6%) was significantly higher than that of non-frontal origin (64.1%) (*P* = 0.038). The incidence of 1p/19q co-deletion in oligodendroglial tumors of the temporal origin (50.0%) showed a trend to be lower than that of non-temporal origin (78.8%), but the difference did not reach significance (*P* = 0.082). For oligodendroglial tumors, however, the incidences of 1p/19q co-deletion between different sides of the brain (76.9%, 68.6% and 100.0%, respectively) were not significantly different.

In 71 oligodendroglial tumors (Grade II and III), the mean global incidence of IDH1/2 mutation was 81.7%. The incidences in Grade II and Grade III (81.8% and 76.5%, respectively) were not significantly different (*P* = 0.892). The incidence of IDH1/2 mutation in oligodendroglial tumors of the frontal origin (92.7%) was significantly higher than that of non-frontal origin (66.7%) (*P* = 0.005). The incidence of IDH1/2 mutation in oligodendroglial tumors of the temporal origin (40.0%) was significantly lower than that of non-temporal origin (88.5%) (*P* = 0.001). For oligodendroglial tumors, however, the incidences of IDH1/2 mutation between different sides of the brain (84.8%, 75.8% and 100.0%, respectively) were not significantly different.

In 150 astrocytic tumors (Grade II, III and IV), the mean global incidence of 1p/19q co-deletion was 12.7%. The incidences in Grade II, III and IV (12.1%, 12.9% and 13.2%, respectively) were not significantly different (*P* = 0.983). Astrocytic tumors did not show different incidences of 1p/19q co-deletion with respect to tumor location. For astrocytic tumors, the incidences of 1p/19q co-deletion between different sides of the brain (15.4%, 10.8% and 9.1%, respectively) were not significantly different either.

In 105 astrocytic tumors (Grade II and III), the mean global incidence of IDH1/2 mutation was 46.7%. The incidences in Grade II and III (51.4% and 36.6%, respectively) were not significantly different (*P* = 0.129). The incidence of IDH1/2 mutation in astrocytic tumors of parietal origin (0.0%) was significantly lower than that of non-parietal origin (51.0%) (P = 0.003). For astrocytic tumors, the incidences of IDH1/2 mutation between different sides of the brain (42.6%, 54.2% and 30.0%, respectively) were not significantly different.

## Discussion

In the present study, we reviewed a series of 528 cases of gliomas retrospectively and compared regional constituents of pathological subsets, regional incidences of 1p/19q co-deletion and IDH1/2 mutation. This study provided a detailed incidence map of glioma subsets (relative), 1p/19q co-deletion and IDH1/2 mutation cross the brain in Chinese patients. The results revealed that gliomas of frontal, temporal and insular origins had significantly different pathological constituents of tumor subsets (*P*<0.001). We also showed that gliomas of the frontal lobe had significantly higher incidence of 1p/19q co-deletion (50.4%) and IDH1/2 mutation (73.5%) than those of non-frontal origin (27.0% and 48.5%, respectively), while gliomas of the temporal origin have significantly lower incidence of 1p/19q co-deletion (23.9%) and IDH1/2 mutation (41.7%) than those of non-temporal origin (39.9% and 63.2%, respectively). Subgroup analysis also confirmed these findings in oligoastrocytic and oligodendroglial tumors. These findings revealed preferential distribution of pathological subsets, 1p/19q co-deletion, and IDH1/2 mutation and implied the distinctiveness among different brain lobes.

The most important finding of our study is the preferential distribution of IDH1/2 mutation in the frontal lobe (73.5%) instead of the temporal lobe (41.7%). This finding was also confirmed in subgroup analysis. Although Lai reported a striking predominance of frontal lobe involvement of primary GBM with IDH1 mutation [25], we provided a more detailed incidence map of IDH1/2 mutation in diffuse gliomas (WHO grade II and III) cross the brain and revealed the preferential distribution of IDH1/2 mutation. The distribution of IDH1/2 mutation resembles that of 1p/19q co-deletion and provided another evidence for the distinctiveness of gliomas from different brain lobes.

In our study, we found that gliomas of different lobes had different constituents of pathological subsets, especially in frontal, temporal, and insular lobes. For example, gliomas of frontal origin were more likely to be oligodendroglioma and less likely to be astrocytoma and glioblastoma than those of non-frontal origin. Gliomas of the temporal origin were more likely to be glioblastoma and less likely to be oligodendroglioma and oligoastrocytoma than those of non-temporal origin. Gliomas of insular origin were more likely to be astrocytoma and oligoastrocytoma and less likely to be glioblastoma and anaplastic astrocytoma than those of non-insular origin. For the first time, we found that gliomas of the temporal origin were more likely to be glioblastomas. Furthermore, we found that low-grade gliomas were preferentially located in the insula, as was suggested by Duffau [19]. These results implied that astrocytic precursor cells were preferentially located in the temporal lobe or that the regional microenvironment in the temporal lobe facilitated the genesis of astrocytic tumors. It also implied that the oligodendroglial precursor cells were preferentially located in the frontal lobe or that the frontal microenvironment facilitated the genesis of oligodendroglial tumors. The preferential distribution of pathological subsets implies correlation between glioma subsets and locations, which will facilitate further investigation of glioma initiation.

According to our results, gliomas of the frontal origin were more likely to harbor prognostic 1p/19q co-deletion than those of non-frontal origin (P<0.001), while gliomas of the temporal origin were less likely to harbor this genetic signature than those of non-temporal origin. In our study, subgroup analysis confirmed the finding that the incidence of 1p/19q co-deletion was higher in the frontal lobe and lower in the temporal lobe in both oligodendroglial and oligoastrocytic tumors. Compared with previous reports, our report provided a more detailed incidence map of 1p/19q co-deletion across the brain in a much larger sample size (Table 2). Zlatescu et al. found that tumors had allelic loss of chromosomes 1p and 19q in anaplastic oligoastrocytoma occurred most frequently in the frontal lobes (53%) and having a tendency for widespread growth across the midline [20]. In Mueller's report, the frequency of 1p/19q co-deletion in the temporal lobe (23.1%) was less than that in non-temporal lobes (81.7%) in oligodendroglial tumors and the frequency in the temporal lobe (33.3%) was less than that in non-temporal lobes (71,4%) in oligoastrocytic tumors [21]. Besides, Huang et al. found that oligodendrogliomas located in the non-temporal lobes were significantly more likely to harbor 1p/19q co-deletion than tumors arising in the insular, temporal lobe, and temporal with another lobe [22].

**Table 2:** Regional molecular heterogeneity about chromosome 1p/19q and IDH1/2 in gliomas reported in English literature.

Authors	Sample size	Findings
Zlatescu et al [20]	N = 64	Anaplastic oligoastrocytomas located in the frontal, parietal, and occipital lobes (65.6%) were significantly more likely to harbor allelic loss of chromosome arms 1p and 19q than histologically indistinguishable tumors arising in the temporal lobe, insula, and diencephalon (34.4%) (*P*<0.001).
Mueller et al [21]	N = 203	The frequency of 1p/19q co-deletion in the temporal lobe (23.1%) were less than that in non-temporal lobes (81.7%) in oligodendroglial tumors (*P*<0.001) and the frequency in the temporal lobe (33.3%) were less than that in non-temporal lobes (71.4%) in oligoastrocytic tumors (*P* = 0.004).
Laigle-Donadey et al [30]	N = 158	Oligodendrogliomas with chromosome 1p deletion was located preferentially in the frontal lobes as compared with the temporal, parietal, and occipital tumors.
Huang et al [22]	N = 105	The combination of LOH 1p and LOH 19q occurred in 26 of 44 (59.1%) non-temporal oligodendroglial tumors, and only 5 of 26 (19.2%) oligodendroglial tumors involving the insular, temporal, and temporal with another lobe (*P* = 0.001). The combination of LOH 1p and LOH 19q occurred in 14 of 26 (53.8%) non-temporal oligoastrocytic tumors, and only 4 of 16 (25%) oligoastrocytic tumors involving the insular, temporal, and temporal with another lobe (*P* = 0.067).
**Present report**	**N = 339**	Gliomas of frontal origin have significantly higher incidence of 1p/19q co-deletion (50.4%) and IDH1/2 mutation (73.5%) than those of non-frontal origin (27.0% and 48.5%, respectively) (*P*<0.001), while gliomas of temporal origin have significantly lower incidence of 1p/19q co-deletion (23.9%) and IDH1/2 mutation (41.7%) than those of non-temporal origin (39.9% and 63.2%, respectively) (*P* = 0.013 and *P* = 0.003, respectively). Subgroup analysis confirmed these findings in oligoastrocytic and oligodendroglial tumors (WHO II and III), respectively. Although the difference of 1p/19q co-deletion in temporal oligodendroglial tumors was marginally significant (*P* = 0.082).

Our study demonstrated a high incidence of 1p/19q co-deletion in insular oligoastrocytic and oligodendroglial tumors (45.0% and 90.0% respectively). The incidence of 1p/19q co-deletion in gliomas of the insular origin was not significantly different from that of non-insular origin, as far as astrocytic, oligoastrocytic or oligodendroglial tumors were concerned, respectively. It was disputable that gliomas in the insular lobe had a lower incidence of 1p/19q co-deletion. Goze et al. [23] studied co-deletion of 1p/19q in a series of 12 Grade II gliomas, including 11 oligodendrogliomas and 1 oligoastrocytoma, in which they found no complete deletion of 1p/19q in them. However, Wu et al. [24] analyzed a series of insular oligodendroglial tumors (n = 14) and found that the frequencies of co-deletion of 1p/19q were 50% in oligodendrogliomas (Grade II and III, n = 8) and 67% in oligoastrocytomas (Grade II and III, n = 6) [24]. Our study with the largest series of patients demonstrated high frequencies of 1p/19q co-deletion in oligodendroglial (90.0%, n = 10) and oligoastrocytic tumors (45.0%, n = 20) of the insular origin. Thanks to the preferential distribution of low-grade gliomas in the insular lobe with a common incidence of 1p/19q co-deletion, favorable outcomes can be expected as reported by Sanai et al. [28]. However, complicated insular anatomy challenges surgical strategies of neurosurgeons [29].

However, selection bias in the present study might exist in this series of glioma patients surgically treated, as the outpatients were not included in the analysis. All the patients were selected for surgery according to the indication and the self-decision of patients, so the composition of tumor types and grades presented here may not be consistent with general frequencies of glioma subsets, which is a weakness of our study.

Besides, problems with assigning tumors to specific brain regions exist in a few cases. In these cases, tumor invading more than one lobe was assigned to the lobe, where the bulk resided, as was described in the material and methods. In 34 tumors involving both frontal and parietal lobes, the ratio of volume was approximately 1∶1, so they were assigned to the frontoparietal region. In spite of these weaknesses, our results and conclusions about constituents of tumor subsets, incidences of 1p/19q co-deletion and IDH 1/2 mutation are convincing and reliable.

In conclusion, by comparing regional constituents of pathological subsets, incidences of 1p/19q co-deletion and IDH1/2 mutation in Chinese patients with gliomas, preferential distribution of tumor subsets, 1p/19q co-deletion and IDH1/2 mutation was confirmed in certain brain regions, implying their distinctiveness in tumor genesis and predictive value for prognosis in Chinese patient populations.
